# Basic Studies Aiming at *Orius minutus* (Hemiptera: Anthocoridae) Mass-Rearing

**DOI:** 10.3390/insects13010077

**Published:** 2022-01-10

**Authors:** Hye-Jeong Jun, Kyoung-Su Kim, Eun-Hye Ham

**Affiliations:** 1 Institute for Bioresources, Osangkinsect Co., Ltd., Guri 11921, Korea; white8962@naver.com; 2 Division of Bioresource Sciences, College of Agriculture and Life Sciences, Kangwon National University, Chuncheon 24341, Korea; kims@kangwon.ac.kr

**Keywords:** artificial oviposition substrate, iron-coated brine shrimp eggs, *Orius minutus*, plant-free rearing, zoophytophagy

## Abstract

**Simple Summary:**

The rapid growth of the biocontrol market is increasing the research into the usefulness of native natural enemies. For the commercial use of native natural enemies, economical mass-rearing technology must be developed. This study was conducted to establish a mass-rearing technique for improving the usability of *Orius minutus* in Korea. This species uses plants for moisture intake, nutritional supplementation, and oviposition substrates. However, the use of plants in mass production systems significantly increases the cost of production. Eggs of stored grain insect pests used as food in rearing *Orius* spp., are the most nutritionally balanced food source, but their high market price necessitates the selection of an economical food source to replace them. In this study, *Ephestia cautella* eggs and iron-coated brine shrimp eggs were selected as diet, and cork sheets were selected as an artificial oviposition substrate to obtain basic data for establishing a plant-free rearing system. The plant-free model developed in our study can reduce rearing costs by 70.5% compared to the conventional mass-rearing model.

**Abstract:**

This study presented biological and economic data for the mass-rearing of *Orius minutus* in Korea. Simplifying the mass-rearing process through an alternative diet and an artificial oviposition substrate is a prerequisite for enhancing the usability of this insect as a biological control agent. We compare the hatch rate of *O. minutus* eggs deposited on a plant substrate with that of eggs deposited on two artificial substrates, cork sheets and rubber. The results indicate that cork sheet is the most cost-effective artificial oviposition substrate for the mass-rearing of *O. minutus*. We also examine five feeding treatments that included two types of brine shrimp eggs and eggs of *Ephestia cautella* to compare the number of eggs laid in the fifth generation. We found no significant difference between the two treatment groups; 61.3 eggs were laid in the treatment group fed iron-coated brine shrimp and moth eggs, and 67.4 eggs were laid in the control group. The plant-free model developed in our study can reduce rearing costs by 70.5% compared to the conventional mass-rearing model.

## 1. Introduction

The commercial use of biological control has developed quickly and created the need for advanced rearing techniques, cost-effective substitute diets, and reliable methods of field application to improve the utilization of biological control agents (BCAs) [1,2]. According to van Lenteren et al. [3], approximately 500 commercial producers world-wide supply about 350 species of BCAs. About 30 of them are mass producers, and about 20 are located in Europe [4]. In the international biological control market, the top 25 BCAs originate from Europe and are imported for domestic use [4]. Imported foreign species are associated with an ecological risk of eliminating native species or reducing the population in a specific region, possibly changing the entire biological community [5,6]. Thus, the development of indigenous-insect resource technologies is imperative for the preservation of biodiversity and sustainable use.

The polyphagous predators, *Orius* spp. (Hemiptera: Anthocoridae), came into use with *O. insidiosus* (Say) in 1985; various product species, such as *O. laevigatus* (Fieber) and *O. strigicollis* (Poppius), developed in 1993 and 2000, respectively, are now widely used for controlling insect pests [7,8,9]. According to van Lenteren [4], *O. laevigatus* belongs to the group of 12 most economically important BCAs, and it is being increasingly used. *O. laevigatus* is also one of eight BCAs registered with the National Agricultural Products Quality Management Service in Korea [10]. Although the first efforts to use natural predators of pests occurred more than 20 years ago, a system-level connection of breeding-circulation-consumption is scarce [11]. More than 35 BCAs are imported to Korea for use in biological control [12].

The present study was conducted to establish a mass-rearing technique for improving the usability of *Orius minutus* (L.), an indigenous Asian species that is expected to show effective control in agriculture, because it is already adapted to domestic environments. Previous studies reported the potential of *O. minutus* as a biological control of aphids, leaf mites, and diamondback moths (*Plutella xylostella* (L.) (Lepidoptera: Plutellidae)) [13,14,15,16]. We also verified the predatory ability of the two-spotted spider mite (*Tetranychus urticae* Koch (Acari: Tetranychidae)), which was 1.39 times higher than that of *O. laevigatus* [17]. Other studies have examined reproductive diapause due to photoperiod [18], field applications [19], ecological characteristics [20], temperature-dependent growth characteristics [21], sex pheromones [22], and mating and morphological characteristics [23]. However, few studies of mass-rearing have been conducted [24].

*Orius* spp. is zoophytophagous, feeding on a variety of prey, plants, and plant products, and takes advantage of plants for moisture intake, nutrition supplements, and oviposition substrates [25]. Thus, the need for researching substitute oviposition substrates has been discussed [26,27,28]. The elimination of plant materials may help rationalize the production of *Orius* spp. However, some studies focused on substituting less common hosts with more common plants such as green beans, kidney beans (*Phaseolus vulgaris* L.), and peppers (*Capsicum annuum* L.) [29,30]. Castañe and Zalom [31] used gelatin coated with parafilm as an oviposition substrate for *O. insidiosus*. De Puysseleyr et al. [30,32] employed a parafilm dome (1.5 cm diameter, 1 cm high) utilizing a diet encapsulation device as an oviposition substrate for *Nesidiocoris tenuis* Reuter (Heteroptera: Miridae) and *O. laevigatus*. The parafilm layer must be cut as soon as the eggs hatch to allow the nymphs to escape. However, most of the nymphs stick to the surface of the parafilm and cannot escape. Therefore, although the parafilm can be cut manually in small-scale rearing, this technique is impractical for a large-scale rearing system [30].

In insect rearing, food sources have a significant impact not only on normal growth and healthy reproduction, but also on product price formation [33,34]. Eggs of stored grain insect pests, such as *Ephestia kuehniella* (Zeller) (Lepidoptera: Pyralidae) and *E. cautella* (Walker), used as food in rearing *Orius* spp., are the most nutritionally balanced food source, but their high market price necessitates the selection of an economical food source to replace them [35,36]. Artificial feeding techniques using a liver and egg yolk combination, the brine shrimp *Artmia franciscana* Kellogg (Anostraca: Artemiidae), and a whole-pupa homogenate of the *Antheraea paphia* (L.) (Lepidoptera: Saturniidae) have been reported [28,29,30,37,38]. Among them, brine shrimp has been studied abroad as an alternative food for various insects, such as *Harmonia axyridis* (Pallas) (Coleoptera: Coccinellidae) [39], *O. laevigatus* [37], *Iphiseius degenerans* (Berlese) (Acari: Phytoseiidae) [40], and *Coleomegilla maculata* De Geer (Coleoptera: Coccinellidae) [41], but it has not yet been studied in Korea. Arijs and De Clercq [37] confirmed that when brine shrimp cysts dried at 50 °C for 24 h were supplied to *O. laevigatus*, ecological characteristics such as the preoviposition period, lifespan, and hatching rate were similar to the control group in which *E. kuehniella* eggs were supplied.

This study was conducted to stabilize mass-rearing *O. minutus* through alternative feeds and artificial oviposition substrates. We selected an artificial oviposition substrate as a substitute for plants in order to simplify the production process and establish an economical mass-rearing system. Furthermore, we performed an economic analysis and confirmed a reduction in costs when our model is compared with the conventional mass-rearing model.

## 2. Materials and Methods

### 2.1. Insect and Experimental Conditions

*O. minutus* adults that developed on *Metasequoia glyptostroboides* Hu and W.C. Cheng (Pinales: Cupressaceae), in Osan, Gyunggi-do, in 2016, were received by Gyeonggi-do Forest Environment Research Center and used for the experiments. The insects were reared in a 10 L container (22 cm diameter, 32.6 cm height, Newpack Co., Ltd., Pocheon, Korea) with a ventilation mesh attached on one side (13 × 15 cm). Frozen eggs of *E. cautella*, a food source, and organic *Sedum sarmentosum* Bunge (Saxifragales: Crassulaceae), the plants for oviposition, were replaced every other day. The rearing and experiments were conducted in the laboratory under conditions of 25 ± 1 °C, at 60 to 75% relative humidity and a 16L:8D photoperiod.

### 2.2. Effect of Moisture on Development

Six different treatments were compared to assess the effect of the moisture source on the development of *O. minutus*. In the first to third treatments, the stems of *S. samentosum* were cut at 2 cm intervals and used. In the second to fifth treatments, 0.4 mL of water or honey-water was supplied on filter paper using a pipette. No moisture source was provided in the sixth treatment (control). Newly hatched nymphs (<24 h old) from the laboratory culture were transferred individually to insect-breeding dishes (5 cm diameter, 1.5 cm height, 310050, SPL Life Science Co., Ltd., Pocheon, Korea) with a mesh-screened vent hole in the lid (1 cm diameter) (*n* = 15). Each dish contained a filter paper (5 cm diameter) on the bottom, and 200 frozen eggs of *E. cautella* were provided every other day. Moisture sources were replaced every day. The developmental time to reach the adult stage and the nymph survival rate were recorded.

### 2.3. Selection of Artificial Oviposition Substrates

We selected cork sheets (30 × 20 × 3 cm, E612, Bluewin Co., Ltd., Paju, Korea) and rubber bands (1.3 × 7 × 1.3 cm, Hyup Jin Company, Seoul, Korea) as candidate oviposition substrates for *O. minutus.* The fecundity and egg hatch on cork sheet and rubber were compared with plants (stems of *S. samentosum* cut at 2 cm intervals). The cork sheets were cut into squares (1.5 × 1 × 0.5 cm) and placed on the bottom of insect-breeding dishes (10 cm diameter, 4 cm height, 310102, SPL Life Science Co., Ltd., Korea). Each dish had a ventilation mesh (4 cm diameter) on the lid and was provided with diet (frozen eggs of *E. cautella*) and a kitchen towel (580517, Georgia-Pacific Corporation, Atlanta, GA, USA) soaked with honey water. The kitchen towel was cut into squares (18 × 14 cm) and folded into small pieces (4.5 × 3.5 cm). Three pairs of adults (10 days post emergence) were placed in each dish, and their fecundity was observed under a microscope after 24 h. In total, 120 pairs of adults were used in each experiment. The hatching rate was recorded after transferring the counted eggs to a breeding dish (5 cm diameter, 1.5 cm height, 310050, SPL Life Science Co., Ltd., Pocheon, Korea) for five days and was compared to that of plants.

### 2.4. Oviposition Substrate Preference Using Cork Sheets

We examined both the thickness and the texture of a cross-section of cork sheets—selected as a substitute for live plants—using the choice test. To examine thickness, 1-mm, 3-mm, and 5-mm thick cork pieces were cut from a 5-cm × 0.5-cm corkboard (E712, E612, E912, Bluewin Co., Ltd., Paju, Korea). The cork pieces and five pairs of adults (10 days post emergence) were placed in the breeding dish (10 cm diameter, 4 cm height, 310102, SPL Life Science Co., Ltd., Pocheon, Korea) with a ventilation mesh (4 cm diameter) on the lid. The number of eggs was observed under a microscope (Dimis-ME, SIWON, Anyang, Korea) after 24 h. In total, 60 pairs of adults were used in each experiment.

To examine texture, the experiment was conducted using the same breeding dish and the same methods as above, and the top of the cork (5 cm in length) was smooth, whereas the sides were rough (*n* = 12). Because few eggs were oviposited on 1-mm thick cork pieces in the thickness experiment, we excluded these pieces from the texture experiment.

### 2.5. Hatch Rate and Moisture Supply of Artificial Oviposition Substrate

We examined methods to improve the moisture content of the cork sheets, which is dry compared to the moisture-containing plants. An improved moisture content was anticipated to improve the hatch rate. Six treatments were investigated: T1 (cork with wet oasis attached + vermiculite and buckwheat husk with water), T2 (cork + vermiculite and buckwheat husk with water), T3 (cork with wet oasis attached), T4 (cork with oasis attached + water (only after egg-laying)), T5 (cork) and control (plant). Briefly, the treatments were categorized by the attachment of an oasis (6 × 2 × 2 cm, Floral foam, Smithers-Oasis Korea Co., Ltd., Cheonan, Korea), distilled water immersion, or the presence of moisture-retaining material (100 mL of vermiculite, 225 mL of buckwheat shells, and 30 mL of distilled water). For each treatment, three pairs of *O. minutus* adults (8 days post emergence), 200 eggs of *E. cautella*, and a kitchen towel soaked in honey water were placed in the breeding dish (10 cm diameter, 4 cm height, 310102, SPL Life Science Co., Ltd., Pocheon, Korea). After 24 h, the eggs were counted under a microscope and placed in a new breeding dish (5 cm diameter, 1.5 cm height, 310050, SPL Life Science Co., Ltd., Pocheon, Korea). In each treatment, 144–233 eggs were used. The hatch rate was recorded for five days and compared to that of plants.

### 2.6. Effect of Diet on Development and Reproduction

This experiment was conducted to select an economical candidate diet that could replace the eggs of *E. cautella* used as food for *Orius* spp. In a breeding dish (5 cm diameter, 1.5 cm height, 310050, SPL Life Science Co., Ltd., Pocheon, Korea), a newly hatched nymph of *O. minutus* (<24 h old) and 3 cm of *S. samentosum* stems were placed, and the developmental time to reach the adult stage according to the different diets was recorded (*n* = 15–16). In the same way as above, in a breeding dish (10 cm diameter, 4 cm height, 310102, SPL Life Science Co., Ltd., Pocheon, Korea), 10 eggs of *O. minutus* (<24 h old) and 3 cm of *S. samentosum* stems were placed, and the survival rate was recorded (*n* = 167–174). Adult individuals were placed in a breeding dish (10 cm diameter, 4 cm height, 310102) in pairs, and the ratio of egg-laying females and the number of eggs per day were confirmed (*n* = 19–28). The diets consisted of 0.1 g of eggs of *E. cautella* (E), iron-coated brine shrimp eggs (I) (Sep-art^®^, Myungsun Co., Ltd., Seoul, Korea), or granular brine shrimp eggs (G) (Artemia^®^, PSP Co., Yongin, Korea) supplied in each container.

In order to select the optimal food combination, the diet sources (E/E, fed E to nymphs and adults, I/I, fed I to nymphs and adults, G/G, fed G to nymphs and adults, E/I, fed E to nymphs and I to adults, E/G, fed E to nymphs and G to adults, E+I/E+I, fed E+I to nymphs and adults, E+G/E+G, fed E+G to nymphs and adults) were fed up to the fifth generation. The number of eggs laid in the treatment groups for 10 days after the first egg laying commenced was compared with the control group fed *E. cautella* eggs. In each treatment, 150 eggs of *O. minutus* and kitchen towels soaked in honey water were placed in a 5 L round container with ventilation holes (10 × 8 cm) on the side, and different combinations of food were supplied. The second generation was moved to a small dish (12 cm diameter, 8 cm height, 310122, SPL Life Science Co., Ltd., Pocheon, Korea) and provided food and honey-water every two days. The time elapsed from the egg to the adult stage in each treatment group, and the number of eggs laid was investigated (*n* = 17–72).

### 2.7. Cost Analysis of Plant-Free Rearing System

The survival rate, developmental time (egg to adult), fecundity, and hatching rate of the plant-free rearing system constructed through the above experiments were compared with the existing rearing system with plants [24,41]. The experimental method was carried out in the same manner as above (*n* = 25–166). We then derived the costs to produce 40 bottles (500 adults per bottle) of *O. minutus* using the plant-free rearing system and compared the costs to those of the conventional mass-rearing system.

### 2.8. Statistical Analysis

The results were analyzed using a one-way ANOVA test [42]. Additionally, the oviposition substrate preference of *O. minutus* and the effect of diet on development and reproduction were analyzed using a two-way ANOVA test. Statistical differences of the resulting values were assessed using the Tukey’s Studentized Range test (Type I error = 0.05).

## 3. Results

### 3.1. Effect of Moisture on Development

The nymphs in the control group (no moisture source) experienced 0% survival (Table 1). No statistically significant differences in the developmental time were observed in the treatment groups (*df* = 4,10, *F* = 3.46, *p* = 0.0506). A high survival rate of 86% or more was confirmed in all treatment groups (*df* = 5,12, *F* = 33.47, *p* < 0.0001).

### 3.2. Selection of Artificial Oviposition Substrates

Table 2 compares the daily average number of eggs in each of three treatments: cork, rubber, and plants, the latter being the natural oviposition material. The cork treatment, unlike the rubber band treatment, was confirmed to be statistically similar to plants (*df* = 2,9, *F* = 8.79, *p* = 0.0077). Although egg laying by *O. minutus* was confirmed in the cork, no individual hatching occurred in the cork kept in an extremely dry condition (*df* = 2,11, *F* = 16,970.8, *p* < 0.0001).

### 3.3. Oviposition Substrate Preference Using Cork Sheets

**Figure 1:**
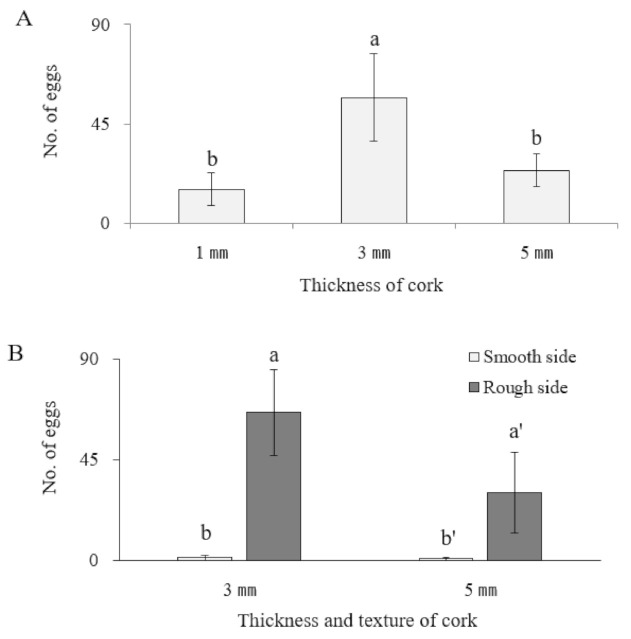
Oviposition preference of *Orius minutus*. (**A**) The analysis was conducted using 1-mm, 3-mm, and 5-mm thick cork substrates. (**B**) Thickness and texture were analyzed together using 3-mm and 5-mm thick, smooth and rough cork substrates. In total, 60 pairs of adults were used in each experiment. The different letters on the standard error bars indicate significant differences between means at Type I error = 0.05.

**Figure 2:**
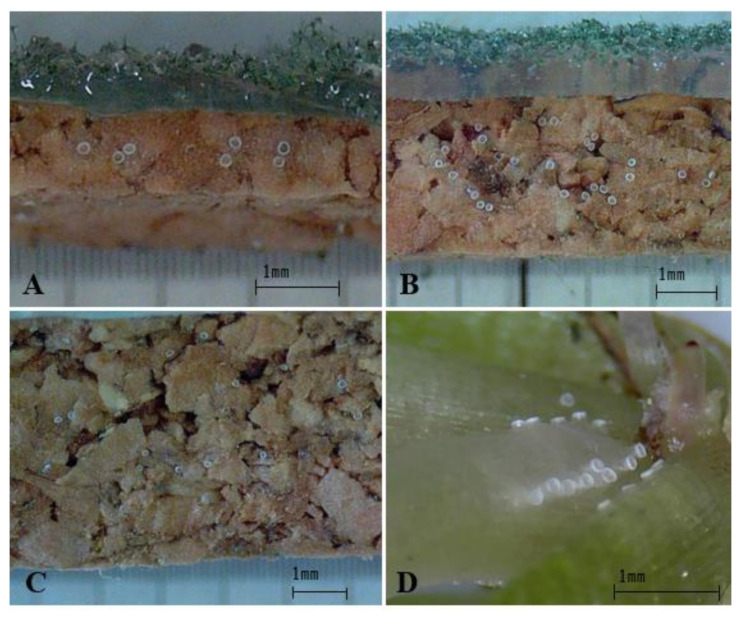
Eggs of *Orius minutus* on rough cork substrates of different thickness ((**A**) 1 mm, (**B**) 3 mm, (**C**) 5 mm) and eggs on plant substrate (**D**).

**Figure 3:**
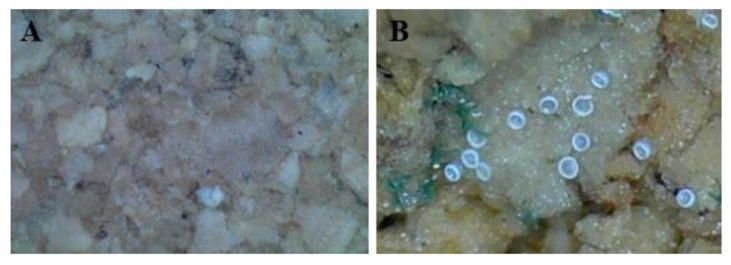
Smooth cork substrate without eggs of *Orius minutus* (**A**) and rough cork substrate with eggs (**B**).

The results of the experiments to determine oviposition substrate preference based on cork thickness and texture are displayed in Figure 1. Based on the number of eggs laid, *O. minutus* preferred the cork sheet of 3-mm thickness for oviposition (Figure 2) (*df* = 2,6, *F* = 8.61, *p* = 0.0173). When offered the same cork thickness with variable texture, *O. minutus* preferred ovipositing on a rough surface rather than a smooth surface (Figure 1, Figure 3) (3-mm: *df* = 1,6, *F* = 7.99, *p* = 0.03; 5-mm: *df* = 1,6, *F* = 10.8, *p* = 0.0117). An interaction between thickness and texture was noted, with *O. minutus* preferring the 3-mm thick rough cork over the 5-mm thick rough cork (thickness: *df* = 1,12, *F* = 7.68, *p* = 0.0169; texture: *df* = 1,12, *F* = 51.77, *p* < 0.0001; thickness × texture: *df* = 1,12, *F* = 7.26, *p* = 0.0195).

### 3.4. Hatch Rate and Moisture Supply of Artificial Oviposition Substrates

**Figure 4:**
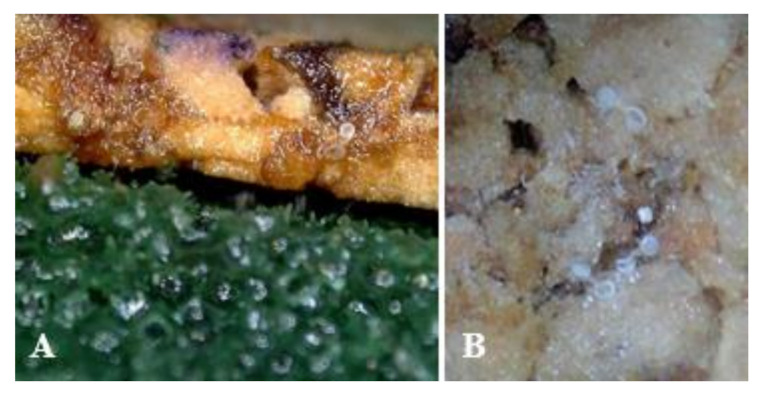
Wet rough cork with oasis attached (**A**) and hatched eggs of *Orius minutus* on rough cork substrate with oasis attached (**B**).

Table 3 shows six treatments used to examine the effects of moisture on the hatch rate. The treatment where an oasis and moisture-retaining material (vermiculite and buckwheat husk with water) were used to improve moisture showed a high hatch rate (*df =* 5,18, *F* = 211.60, *p* < 0.0001) that was not statistically different from the hatch rate on plants. The hatch rate was 72.8% in the mixed treatment of wet oasis and vermiculite and buckwheat husk with water, which is higher by 5.8% than that in the treatment with oasis only, but this difference is not statistically significant. The treatment where a wet oasis was attached to the cork throughout the entire developmental time of *O. minutus* eggs also showed a high hatch rate that was not statistically different from the hatch rate on plants (see Figure 4 for photos of cork with oasis attached and hatched eggs).

### 3.5. Effect of Diet on Development and Reproduction

To select an alternative diet for *O. minutus*, the developmental time and survival rate were recorded while supplying candidate diets (Table 4). We confirmed that the group treated with brine shrimp eggs had a longer developmental time, of 5.9–7.8 days, compared to other treatment groups (*df* = 2,6, *F* = 27.5, *p* = 0.01). The survival rate of the treatment group fed iron-coated brine shrimp eggs was 67.5%, which was 5.9 times higher than that of granular brine shrimp eggs, confirming the statistically significant results (*df* = 2,6, *F* = 233.16, *p* < 0.0001). In the fecundity experiment, the treatment group fed granular brine shrimp eggs produced no females to lay eggs, and therefore the fecundity could not be assessed. The fecundity in the groups fed eggs of *E. cautella* and iron-coated brine shrimp eggs was high (*df* = 2,6, *F* = 686.02, *p* < 0.0001). The mean number of eggs deposited per day in the E and I treatment groups was similar (*df* = 1,4, *F* = 4.68, *p* = 0.0757).

**Figure 5:**
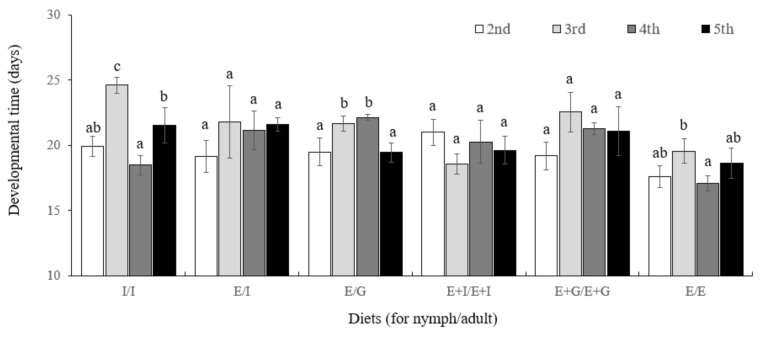
Mean developmental time (days) of *Orius minutus* by generation (2nd, 3rd, 4th, and 5th) and diet type (I/I, fed I to nymphs and adults, E/I, fed E to nymphs and I to adults, E/G, fed E to nymphs and G to adults, E+I/E+I, fed E+I to nymphs and adults, E+G/E+G, fed E+G to nymphs and adults, E/E, fed E to nymphs and adults). Different letters on the standard error bars for each diet category indicate significant differences between means at Type I error = 0.05.

The results of analyzing the growth period by feeding six different diet combinations and breeding them continuously for five generations are shown in the Figure 5. In each treatment group, although a slight difference was observed in the developmental time according to the diet source and generation (I/I: *df* = 4,10, *F* = 2.71, *p* = 0.0915; E/I: *df* = 4,10, *F* = 9.96, *p* = 0.0016; E/G: *df* = 4,10, *F* = 11.45, *p* = 0.0009; I+E/I+E: *df* = 4,10, *F* = 4.65, *p* = 0.0222; G+E/G+E: *df* = 4,10, *F* = 22.69, *p <* 0.0001), as a result of a two-way analysis, no statistically significant difference was identified (diet source: *df* = 5,48, *F* = 0.92, *p* = 0.4788; generation: *df* = 3,48, *F* = 1.34, *p* = 0.2721; diet source × generation: *df* = 15,48, *F* = 0.84, *p* = 0.6011).

In the intergenerational fecundity experiment, the treatment group fed granular brine shrimp eggs produced only one adult due to cannibalism, and therefore the fecundity could not be assessed. In the two treatment groups supplied with a mixture of *E. cautella* eggs and two types of brine shrimp eggs, the fecundity up to the fourth generation was similar to that of the control group supplied with *E. cautella* eggs (1st: *df* = 5,17, *F* = 50.54, *p* < 0.0001; 2nd: *df* = 5,17, *F* = 32.91, *p* < 0.0001; 3rd: *df* = 5,17, *F* = 11.34, *p =* 0.0003; 4th: *df* = 5,17, *F* = 50.90, *p* < 0.0001; 5th: *df* = 5,17, *F* = 89.25, *p* < 0.0001) (Table 5). The mean fecundity was found to be statistically different by diet source and generation (diet source: *df* = 5,60, *F* = 89.24, *p <* 0.0001; generation: *df* = 4,60, *F* = 4.01, *p* = 0.006; diet source × generation: *df* = 20,60, *F* = 3.12, *p* = 0.0003).

### 3.6. Cost Analysis of the Plant-Free Rearing System

**Figure 6:**
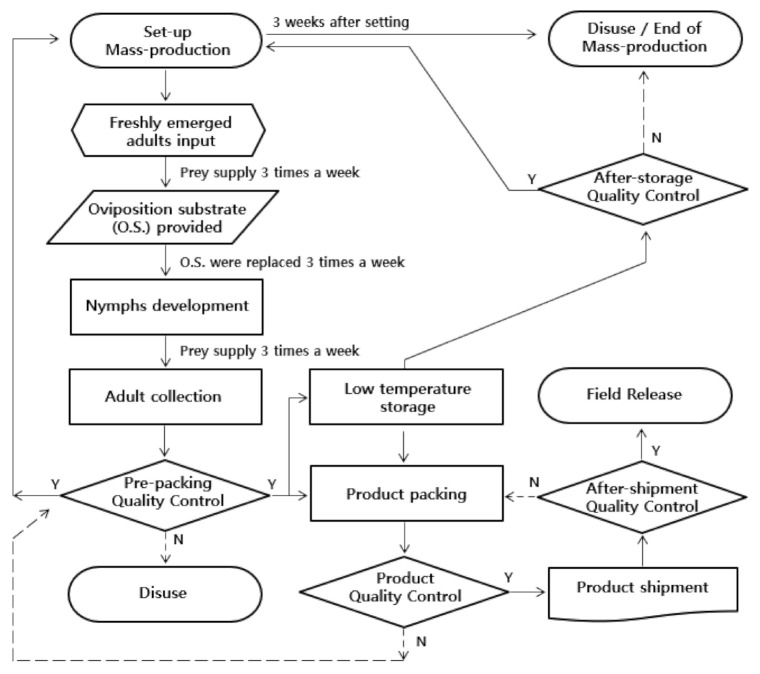
Flow chart of mass-rearing of *Orius minutus.* Y means passing the quality control result, and *n* means failing the quality control result.

Both the plant-free rearing system and the conventional mass-rearing system using plants showed similar results in survival rate (*df* = 1,5, *F* = 0.42, *p =* 0.5516), developmental time (*df* = 1,5, *F* = 1.13, *p =* 0.3481), fecundity (*df* = 1,5, *F* = 0.66, *p =* 0.4630), and hatching rate (*df* = 1,7, *F* = 0.27, *p =* 0.6193) (Table 6). In this study, we established a plant-free rearing system by selecting cork sheets as an oviposition substrate for *O. minutus*, a predatory insect that normally oviposits on plants (Figure 6). A cost analysis of our proposed system was performed and compared to the conventional mass-rearing system (Table 7). We derived the egg-harvesting cost of the conventional mass-rearing model by referring to the “industrial standards of insect breeding and specification II,” published in the Rural Development Administration [43] and Korea Forest Service [24], for the production method for *O. laevigatus*. Compared with the conventional mass-rearing model, the egg-harvesting cost was reduced by 98.2% and the diet cost by 48.3%, thereby reducing the total production cost by 70.5%.

## 4. Discussion

In this study, we presented biological and economic data for the development of *O. minutus*, an indigenous insect species, using a plant-free rearing system for the localization of biological control agents, which are increasingly dependent on imports.

Insects are known to replenish moisture by taking in fluids from the xylem of plants [44]. According to a study by Bonte and De Clercq [25], insects can grow successfully without plants if other sources are available to replenish moisture and nutrients, which was also confirmed in this study (Table 1).

The establishment of mass-rearing techniques to manage natural predators stably is a crucial element in biological control [45,46]. This study investigated the preference of artificial oviposition substrates in *O. minutus*; substrate preference based on thickness and texture; and hatch rate related to the moisture supply. The artificial substrate preferred by *O. minutus* is a 3-mm-thick cork sheet with a rough texture (Figure 1, Figure 2 and Figure 3). The oviposition by *O. minutus* was confirmed in the cork sheet, but typical hatching was not probable in the chemically stable cork sheet because the water could not seep through (Table 2). Delobel [47] reported that maintaining a low humidity reduces the metabolic activities of eggs of *Atherigona soccata* Rondani (Diptera: Muscidae), prolonging the egg stage and leading to a failure in hatching. When *Delia radicum* (L.) (Diptera: Anthomyiidae) eggs were exposed to a 20–29 °C temperature and 5–100% relative humidity, Lepage et al. [48] reported that the temperature did not affect survival significantly, whereas humidity caused egg mortality. Bonte and De Clercq [25] reported that the moisture supply affects the development and breeding of *O. laevigatus* greatly in indoor rearing. In our study, a low hatch rate of 34.7% was recorded for the mixed treatment of vermiculite and buckwheat husk with water and cork sheet, which confirmed that the moisture content external to the cork does not affect the egg hatch inside the cork (Table 3). A high hatch rate (68.8%, 72.8%) similar to the hatch rate on plants (71.6) was observed in the treatment where a wet oasis was attached consistently during the developmental time.

Coll [49] reported that plants are an essential element for breeding—an element for oviposition and a food substitute for development—to *O. insidiosus*, which is zoophytophagous. In this study, we observed a slightly greater average number of eggs with the cork substrate (13.1) than with plants (12.3) (Table 2). Although these averages are not significantly different, we suspect that the honey-water supplied with the cork treatment induced a positive effect. De Puysseleyr et al. [30] reported a successful oviposition by *O. laevigatus* when using wet cotton wrapped with parafilm. Bonte and De Clercq [25] succeeded in breeding 90% of the nymphs into adults with only food substitute and without plants. However, the weight of female and male adults was reduced by 9.1% and 14%, respectively, compared to using food substitute and plants. De Puysseleyr et al. [32] also reported that the weight of the fifth-generation female and male adults decreased by 4% and 6%, respectively, when *Nesidiocoris tenuis* (Reuter) (Hemiptera: Miridae) was bred without plants. Conversely, Cohen and Debolt [50] reported that the presence or absence of plants does not affect adult weight in *Geocoris punctipes* (Say) (Hemiptera: Lygaeidae) breeding. Similarly, in De Puysseleyr et al. [30], a weight reduction of male adult *O. laevigatus* was not observed up to the fourth generation when reared indoors. Weight was slightly reduced for females, but the authors reported no influence on the number of oocytes and eggs. The presence or absence of plants can affect omnivorous insects that are reared indoors differently according to the species and breeding methods, leading to disparate findings. Therefore, further study is necessary to clarify the influence of the plant-free rearing system on the development and continual breeding of *O. minutus* [32,50].

For a predator to be able to compete with the main biological control agents in the biological control market, economical mass-rearing techniques have to be established. However, the need for host plants or natural food for breeding can more than double production costs compared to those of the species produced with food substitutes and without plants [46]. In the case of conventional *Orius* spp. breeding, the eggs of moths supplied as food have a high nutritional value, but also a high production cost [51]. Therefore, in this study, iron-coated brine shrimp eggs, which can replace moth eggs, were selected as an alternative diet for *O. minutus* (Table 4). In the case of granular brine shrimp eggs, the survival rate of *O*. *minutus* decreased due to an increase in cannibalism as compensation for the nutritional deficiency [52,53,54,55]. Nutrient imbalances tend to be expressed after several generations of breeding [51]. Taking this tendency into consideration, seven diet combinations were fed continuously for five generations, and the developmental time and number of eggs were compared and analyzed (Table 5, Figure 5). The number of eggs decreased in all treatment groups fed brine shrimp eggs, which are not coated with iron, up to the fifth generation. We attribute this effect over the generations to the lack of fatty acids in the brine shrimp eggs. De Clercq et al. [51] reported that flour moth eggs were almost three times richer in fatty acids than brine shrimp cysts. Most of the fatty acids consumed by insects are stored in triglycerides as an energy source, and the rest are known to play an important role as phospholipid membrane structures or signaling molecules [56]. De Clercq et al. [51] reported that brine shrimp cysts can be used as supplementary food for the mass production of *O. laviegatus*, but brine shrimp cysts are not suitable for long-term breeding because the developmental time is 18% lower and the number of eggs is 60% lower in the third generation compared to feeding *E. cautella* eggs. Based on our results, mixing *E. cautella* eggs and iron-coated brine shrimp eggs for rearing *O. minutus* will be beneficial in terms of nutrition and economics. Iron is required for oxygen transport and catalytic roles in oxidative metabolism and is essential for living organisms [57]. Many aspects of the iron metabolism in insects remain poorly understood [58]. Tang and Zhou [58] reported the first genetic evidence that secretory ferritin directly participates in dietary iron absorption in *Drosophila melanogaster* Meigen (Diptera: Drosophilieae). Ferritin is considered to be an anti-oxidant protein and the major iron storage site at the molecular level [59]. It has long been hypothesized that ferritin is involved in dietary iron absorption and transport in insects due to the secretory character of insect ferritin [58]. In mammals, the exported iron from enterocytes is mostly bound and transported by serum transferrin (Tsf) [60]. The Tsf delivery of iron via a receptor has not been shown in any insects because no homologue of the mammalian Tsf receptor has been identified so far from the published genome sequences of insects [61,62]. This suggests that insects could have a different class of Tsf receptors or that iron is taken up by an alternate transport mechanism [61]. More research is needed on how *O. minutus* consumes iron and uses it for development and survival.

In this study, *E. cautella* eggs and iron-coated brine shrimp eggs as diet and cork sheets as an artificial oviposition substrate were used to obtain basic data for establishing a plant-free rearing system (Table 6, Figure 6). We calculated the actual cost for the materials that were used in this study and the materials necessary for conventional mass-rearing (Table 7). After adding the cost of the workforce, facility, and equipment for growing plants, we anticipate a reduction of more that 70% in costs with the plant-free rearing model compared to the costs with the conventional model. The “*Orius* spp. plant-free rearing system” proposed in this study has been patented (Patent registration number: 10-2209388).

## Figures and Tables

**Table 1:** The effect of moisture source on the development of *Orius minutus* reared on *Ephestiea cautella* eggs ^1^.

Treatment	Developmental Time (Days)	Nymphal Survival (%)
Plant	11.7 ± 0.6	93.3 ± 9.4
Plant and water dome	11.9 ± 0.99	100.0 ± 0.0
Plant and honey-water dome	12.1 ± 0.4	100.0 ± 0.0
Water dome	12.9 ± 0.1	86.7 ± 9.4
Honey-water dome	12.7 ± 0.1	86.7 ± 18.9
Control (no moisture source)		0

^1^ Values represent means ± standard deviation; *n* = 15 for each treatment. No significant differences were found among the first five treatments for developmental time or nymphal survival (*p* > 0.05).

**Table 2:** Number of oviposited eggs per three pairs of *Orius minutus* and egg hatch (%) on different oviposition schemes ^1^.

Oviposition Substrate	No. of Eggs	Egg Hatch (%)
Cork	13.1 ± 2.4 a	0 b
Rubber band	4.1 ± 4.7 b	0 b
Plant	12.3 ± 4.1 a	71.6 ± 0.9 a

^1^ Values represent means ± standard deviation; *n* = 40 sets of three pairs for each substrate. Values followed by the same letter in each column are not significantly different (*p* > 0.05).

**Table 3:** Hatching rate of *Orius minutus* using six different moisture sources.

Treatment	Oviposition Substrate ^1^	*n* ^2^	Moisture Source ^3^	Egg Hatch (%) ^4^
T1	Cork sheet	149	Oasis, vermiculite, and buckwheat husk with water	72.8 ± 3.6 a
T2	Cork sheet	144	Vermiculite and buckwheat husk with water	34.7 ± 4.6 c
T3	Cork sheet	233	Oasis with water	68.8 ± 4.1 a
T4	Cork sheet	157	Oasis with water (only after egg-laying)	51.5 ± 4.1 b
T5	Cork sheet	213		0 d
Control	Plant	166		71.6 ± 0.9 a

^1^ Cork sheet size: 5 × 0.5 cm. ^2^ *n*: number of eggs per treatment. ^3^ Oasis size: 6 × 2 × 2 cm; volume of vermiculite, buckwheat husk and water: 100, 225, and 30 mL, respectively. ^4^ Values represent means ± standard deviation, values followed by the same letter are not significantly different (*p* > 0.05).

**Table 4:** Developmental time and survival rate experienced by *Orius minutus* under three different diet sources ^1^.

Diet ^2^	Developmental Time (Days)	Survival (%)	% Egg Laying Females	No. of Eggs Deposited per Day
E	12.1 ± 0.4 (15) a	72.7 ± 3.8 (167) a	92.6 ± 6.4 (27) a	3.8 ± 0.7 (25) a
I	14.0 ± 0.2 (15) a	67.5 ± 3.7 (167) a	100.0 ± 0.0 (28) a	4.8 ± 0.2 (28) a
G	19.9 ± 2.2 (16) b	11.4 ± 1.3 (174) b	0 (19) b	-

^1^ Numbers in parentheses refer to the number of individuals per treatment group. Values represent means ± standard deviation, values followed by the same letter are not significantly different (*p* > 0.05). ^2^ E, *Ephestia cautella* egg; I, Iron-coated brine shrimp eggs; G, Granular brine shrimp eggs.

**Table 5:** Mean fecundity of *Orius minutus* by generations and diets ^1^.

Diets (for Nymph/Adult) ^2^	Fecundity (± S.D.) for Ten Days after Egg Laying/Generation				
	1st	2nd	3rd	4th	5th
E/E	62.9 ± 9.3 (27) a	63.7 ± 7.5 (25) a	63.6 ± 12.2 (21) a	68.8 ± 12.7 (17) a	76.9 ± 5.7 (18) a
I/I	36.4 ± 6.2 (64) b	25.3 ± 9.8 (26) b	39.3 ± 7.1 (24) b	27.2 ± 0.9 (48) b	22.4 ± 3.2 (56) c
G/G	-	-	-	-	-
E/I	44.7 ± 3.6 (64) b	36.1 ± 2.8 (66) b	31.6 ± 4.1 (62) b	32.5 ± 3.9 (50) b	21.6 ± 4.1 (70) c
E/G	13.2 ± 2.0 (62) c	21.8 ± 4.2 (62) b	28.8 ± 5.4 (42) b	11.4 ± 2.7 (48) c	9.0 ± 0.6 (64) c
E+I/E+I	69.0 ± 0.2 (70) a	68.7 ± 5.5 (62) a	62.4 ± 4.1 (70) a	60.5 ± 2.3 (62) a	53.5 ± 6.2 (70) b
E+G/E+G	73.9 ± 3.7 (72) a	74.0 ± 2.2 (68) a	44.4 ± 5.5 (66) ab	66.5 ± 2.1 (70) a	63.2 ± 3.4 (22) b

^1^ Numbers in parentheses refer to the number of individuals per treatment group. Values (means ± standard deviation) followed by the same letter on the same column are not significantly different (*p* > 0.05). ^2^ The diets consisted of 0.1 g of eggs of *Ephestia* *cautella* (E), iron-coated brine shrimp eggs (I), or granular brine shrimp eggs (G). (E/E, fed E to nymphs and adults, I/I, fed I to nymphs and adults, G/G, fed G to nymphs and adults, E/I, fed E to nymphs and I to adults, E/G, fed E to nymphs and G to adults, E+I/E+I, fed E+I to nymphs and adults, E+G/E+G, fed E+G to nymphs and adults).

**Table 6:** Comparison of ecological characteristics according to two different rearing systems of *Orius minutus* ^1^.

Treatment	Survival (%)	Developmental Time (Days)	Fecundity/Day	Hatching Rate (%)
Rearing on plants	92.3 ± 4.7 (109) a	19.6 ± 0.6 (109) a	73.1 ± 8.3 (35) a	71.6 ± 0.9 (166) a
Plant-free rearing	90.1 ± 5.1 (33) a	19.2 ± 0.2 (33) a	78.3 ± 7.1 (25) a	72.8 ± 3.6 (149) a

^1^ Numbers in parentheses refer to the number of individuals per treatment group. Values (means ± standard deviation) followed by the same letter are not significantly different (*p* > 0.05).

**Table 7:** Economic analysis of two different oviposition materials to make 40 products of *Orius minutus* (500 pieces per product).

Mass-Rearing Model of RDA (2014) ^1^	Plant-Free Rearing Model
Total Cost $507.42	Total Cost $149.67
- Oviposition Plant-Related Costs = $222.65 Plant (pot): $16.78 × 2 ea. = $33.55	- Oviposition Plant-Related Costs = $4.03 Cork sheet (20 × 30 × 0.3 cm): $1.38 × 2 ea. = $2.77
Soil (50 L): $8.22 × 1 ea. = $8.22	Oasis (23 × 11 × 8 cm): $0.63 × 2 ea. = $1.26
Rearing box (49 × 38 × 8 cm): $3.02 × 2 ea. = $6.04	
Cage (50 × 47 × 37 cm): $83.89 × 2 ea. = $167.77 Compound fertilizer (1 kg): $10.07 × 1 ea. = $10.07	
- Food-related costs ^2^ = $281.86 *Ephestia cautella* egg: $4.70 × 60 g = $281.86	- Food-related costs = $145.65 *Ephestia cautella* egg: $4.7 × 30 g = $140.96 Iron-coated Brine Shrimp eggs: $0.16 × 30 g = $4.68

^1^ Source: Rural Development Administration [43]. ^2^ Source: Korea Forest Service [24].

## Data Availability

The datasets and the images in this study are still being used in ongoing research, they will only be available upon reasonable request.
